# Rheology and Oil–Water Emulsion Stability During Biodegradation of Hydrolyzed Polyacrylamide by *Delftia lacustris* EPDB-8

**DOI:** 10.3390/polym18111268

**Published:** 2026-05-22

**Authors:** Bingjian Sun, Yanshuo Li, Wei Liu, Xin Hu, Shichong Guo, Yiming Li, Jinren Lu, Haoshuai Li, Mutai Bao

**Affiliations:** 1 Frontiers Science Center for Deep Ocean Multispheres and Earth System, Key Laboratory of Marine Chemistry Theory and Technology, Ministry of Education, Ocean University of China, Qingdao 266100, China; 13589885399@163.com (B.S.); huxin@stu.ouc.edu.cn (X.H.);; 2 College of Chemistry and Chemical Engineering, Ocean University of China, Qingdao 266100, China; 3 Key Laboratory of Colloid and Interface Chemistry, Ministry of Education, School of Chemistry and Chemical Engineering, Shandong University, Jinan 250100, China; liuwei1992@sdu.edu.cn

**Keywords:** hydrolyzed polyacrylamide, biodegradation, oil–water emulsion, demulsification, interfacial rheology, produced water

## Abstract

Hydrolyzed polyacrylamide stabilized oil-in-water emulsions are highly persistent because the polymer strengthens both continuous-phase rheology and the oil–water interfacial film, making demulsification difficult in polymer-flooding produced liquids. Here, an hydrolyzed polyacrylamide degrading bacterium, *Delftia lacustris* EPDB-8, was isolated, and its ability to destabilize hydrolyzed polyacrylamide-containing emulsions was investigated from molecular, bulk rheological, and interfacial perspectives. EPDB-8 effectively degraded HPAM, causing marked reductions in total organic carbon, total nitrogen, absolute zeta potential, and polymer molecular weight, with an approximately 63-fold decrease after 7 days. SEM, FT-IR, and GPC analyses showed that biodegradation proceeded through deamidation and random chain scission, collapsing the polymer network and generating low-molecular-weight fragments. Driven by bacterial hydrolyzed polyacrylamide degradation, these structural alterations disrupted the viscoelastic composite interfacial film formed by hydrolyzed polyacrylamide and indigenous surface-active species, directly causing emulsion stabilization to shift from polymer-assisted viscous and steric protection to a less effective asphaltene-dominated interfacial structure and thereby accelerating droplet aggregation, coalescence, and phase separation. Although bacterial cells exerted a transient particle-assisted interfacial effect, long-term emulsion stability remained governed by polymer integrity. This study establishes a mechanistic link between hydrolyzed polyacrylamide biodegradation and the rheological and interfacial evolution governing emulsion breakdown, providing a cost-effective and environmentally benign biological strategy for demulsification and treatment of polymer-flooding produced water. These findings offer practical guidance for the design of microbial-based produced-water treatment systems and contribute to the sustainable management of oilfield wastewater generated during enhanced oil recovery operations.

## 1. Introduction

The management of produced water in the petroleum industry represents one of the most significant environmental and operational challenges in modern energy extraction. As accessible conventional oil reserves decline, the industry has increasingly turned to enhanced oil recovery (EOR) techniques to maximize extraction from mature fields. Among these, polymer flooding using partially hydrolyzed polyacrylamide (HPAM) is widely used [1,2,3]. HPAM is preferentially selected over other EOR polymers due to its exceptional thickening efficiency at low concentrations, cost-effectiveness at industrial scale, and well-established injectability in porous reservoir media. Its partially hydrolyzed structure confers both viscosifying capacity and salt tolerance, making it particularly suitable for the high-salinity, high-temperature conditions encountered in mature oilfields such as those in the Daqing and Shengli basins [4]. However, the very properties that make HPAM effective in the subsurface, specifically its high molecular weight (typically 10^6^ to 10^7^ Da) [5], viscoelasticity, and chemical stability [6], render it a recalcitrant pollutant in the produced water stream (Table 1).

Residual HPAM in produced water stabilizes oil-in-water (O/W) emulsions and reduces the efficiency of conventional separation processes such as gravity settling and flotation [2,3,4,9]. Therefore, understanding how HPAM degradation alters emulsion stability is important not only for wastewater treatment but also for the design of biologically assisted oil–water separation strategies. HPAM adsorbs at the oil-in-water interface through its amide groups (-CONH_2_), and partial hydrolysis introduces carboxylate groups (-COO^−^), which enhance interchain electrostatic repulsion and lead to the extension of polymer chains. The expansion of the polymer chains increases hydrodynamic volume and intermolecular friction, which in turn elevates the apparent viscosity and viscoelasticity of the continuous phase [2,3,12]. Simultaneously, the extended and charged chains form a dense, strongly hydrated adsorption layer on the droplet surfaces. This layer, through steric hindrance and electrostatic repulsion, prevents droplet contact and inhibits coalescence by strengthening the electrical double layer effect [3,4,13]. This increases repulsive interactions and the associated energy barrier, further enhancing kinetic stability. This results in produced water that exceeds discharge regulations for oil content and viscosity [6,7,8,14].

While physical and chemical treatments exist, they are often energy-intensive or generate secondary sludge [7,11,14]. Physical methods, including membrane filtration, adsorption, and UV irradiation, can effectively reduce HPAM concentrations but are limited by membrane fouling, high operational costs, and the inability to mineralize the polymer. Chemical approaches such as Fenton oxidation, ozonation, and electrochemical degradation achieve more complete chain scission through reactive oxygen species yet require substantial chemical inputs, produce toxic byproducts, and face scalability constraints in oilfield settings [11,14,15]. Advanced oxidation processes (AOPs) combining UV/H_2_O_2_ or O_3_/H_2_O_2_ have demonstrated improved degradation efficiency, but their energy demands and reagent costs remain prohibitive for large-volume produced water treatment [14,16]. Consequently, biological treatment has emerged as a promising, sustainable alternative, and substantial effort has been devoted to screening hydrolyzed polyacrylamide-degrading microorganisms from polymer-flooding produced water and elucidating their degradation mechanisms [15,16].

Aerobic biodegradation generally proceeds in a stepwise fashion: amide side groups are preferentially utilized as a nitrogen source, followed by slower cleavage and partial assimilation of the carbon backbone, a sequence that proceeds without detecta-ble formation of neurotoxic acrylamide monomers under tested conditions. Mechanis-tic investigations have implicated amidases, cytochrome P450-type monooxygenases, urease-related enzymes, and peroxidases in the successive decarbonization and deni-trogenation of the polymer [17,18]. More recently, genomic and functional analyses have broadened the known repertoire of degraders, including strains capable of uti-lizing hydrolyzed polyacrylamide as a sole nutrient source. A phylogenetically diverse range of genera has been reported, including Bacillus, Pseudomonas, Klebsiella, Micro-bacterium, Delftia, Agrobacterium, Mycobacterium, Azotobacter, Brevibacillus, and Coryne-bacterium, reflecting the metabolic versatility required to attack this recalcitrant poly-mer [17,18,19,20]. The co-occurrence of these functional guilds in oilfield sludge and pro-duced water suggests that community-level interactions govern in situ degradation rates [6,10,18].

Despite extensive studies on HPAM-degrading microorganisms, the interfacial consequences of HPAM biodegradation remain poorly resolved. In particular, it is still unclear how molecular degradation, charge evolution, and cell-associated adsorption collectively regulate the rheological properties and stability of polymer-containing oil–water emulsions. In this work, we isolated a highly efficient HPAM-degrading strain, *Delftia lacustris* EPDB-8, using stepwise acclimation and evaluated its degradation behavior through TOC, TN, and gel permeation chromatography analyses. More importantly, by integrating scanning electron microscopy, GC-MS, Turbiscan analysis, zeta-potential measurements, and rheological characterization, we examined how biodegradation-induced changes in HPAM structure translate into interfacial evolution and emulsion destabilization. This study aims to establish a mechanistic framework linking microbial HPAM degradation with the breakdown of polymer-supported oil–water interfaces.

## 2. Materials and Methods

### 2.1. Materials

Toluene, *n*-heptane, ethanol, potassium dihydrogen phosphate (KH_2_PO_4_), dipotassium hydrogen phosphate (K_2_HPO_4_), magnesium sulfate heptahydrate (MgSO_4_·7H_2_O), sodium chloride (NaCl), ferrous sulfate heptahydrate (FeSO_4_·7H_2_O), agar, tryptone, yeast extract, and activated alumina were all of analytical grade and purchased from Sinopharm Chemical Reagent Co., Ltd. (Shanghai, China). Hydrolyzed polyacrylamide (HPAM; molecular weight = 2.16 × 10^7^ Da; hydrolysis degree = 10.00%) was obtained from Changan Polymer Co., Ltd. (Dongying, China). Polymer-containing oil sludge was collected from the Daqing Oilfield (Daqing, China) and used as the inoculum source.

All other reagents were of analytical grade. Luria–Bertani (LB) medium, minimal salts medium (MSM) and degradation medium were used for the enrichment, isolation, and domestication of bacteria. LB (L^−1^) contains 10 g tryptone, 5 g yeast extract, and 10 g NaCl. Solid medium was prepared by adding 20 g of agar to LB liquid medium. The MSM (L^−1^) consists of 3.48 g KH_2_PO_4_, 0.7 g MgSO_4_, 1.5 g Na_2_HPO_4_·12H_2_O, 3.96 g (NH_4_)_2_SO_4_ and 0.5 g HPAM. The initial pH of these mediums was adjusted to 7.2. The degradation medium consists of 100 mL of HPAM solution at a concentration of 500 mg/L. All media were sterilized in an autoclaved sterilizer (GI54D, Zealway, Xiamen, China) at 121 °C for 30 min before use.

### 2.2. Isolation and Enrichment of HPAM-Degrading Bacteria

HPAM-degrading bacteria were enriched and isolated from polymer-contaminated sludge collected from the Daqing oilfield. Briefly, 5 g of sludge was added to 100 mL of sterilized LB medium and incubated on a rotary shaker at 25 °C and 150 rpm for 3 days to release microorganisms from the sediment matrix. The culture was then allowed to stand for 20 min to facilitate sedimentation of particulates, and the supernatant was collected as the microbial suspension. Subsequently, 5 mL of the suspension was transferred into 100 mL of fresh sterilized LB medium and incubated under the same conditions for an additional 3 days to further enrich the microbial community. The enriched consortium was then acclimated to HPAM through a stepwise feeding strategy: in the transfer mode, actively growing cultures were periodically transferred into fresh LB medium supplemented with progressively increasing HPAM concentrations; in the accumulation mode, HPAM was incrementally added into the same flask without replenishing the medium, until the HPAM concentration in LB reached 300 mg/L. The final enriched culture was serially diluted (10^−5^, 10^−6^, 10^−7^), and 2 μL of each dilution was spread onto LB solid medium. Plates were incubated at 30 °C and monitored for colony growth. Single colonies were picked and inoculated into LB liquid medium, and isolated strains were stored at −80 °C for further use.

Strain identification was performed based on physiological and biochemical characterization according to Bergey’s Manual of Systematic Bacteriology. 16S rRNA gene sequencing was conducted by BGI Co., Ltd. (Beijing, China). The 16S rRNA gene was amplified using primers 27F and 1492R. The resulting sequences were submitted to the National Center for Biotechnology Information (NCBI) for homology comparison, and a phylogenetic tree was constructed using MEGA 11.0 with the neighbor-joining (NJ) method.

### 2.3. Preparation of Asphaltenes and Resins

Crude oil was extracted from polymer-containing oil sludge (PCOS) by Soxhlet extraction with toluene, then refluxed with *n*-heptane to separate asphaltenes (insoluble residue, redissolved in toluene) from the *n*-heptane-soluble fraction (saturates, aromatics, resins). Resins were isolated from the latter by alumina column chromatography: after *n*-heptane preconditioning, sequential elution with *n*-heptane, toluene, and toluene/ethanol (1:1, *v*/*v*) removed saturates, aromatics, and resins, respectively. The resin eluate was dried under vacuum, and the asphaltene fraction was recovered by toluene evaporation.

### 2.4. Emulsion Preparation

Heptol (toluene/*n*-heptane, 60:40 *v*/*v*) was used as the model oil phase to simulate crude oil systems for investigating interfacial adsorption behavior. Emulsions were prepared by mixing equal volumes (5 mL each) of aqueous and heptol phases in glass vials, followed by homogenization at 12,000 rpm for 2 min using an IKA T100 high-speed disperser (IKA-Werke GmbH & Co. KG, Staufen im Breisgau, Germany). The asphaltene and resin fractions extracted from PCOS were dissolved in heptol at final concentrations of 0.5 g/L asphaltenes and 0.5 g/L resins, unless otherwise stated. The resultant emulsions were allowed to stand quiescently for 6 h prior to subsequent analyses. Control emulsions without bacteria were established based on stabilizer composition. Five control groups (E_A_, E_AR_, E_AH_, E_RH_, and E_ARH_) were set up, where E_A_ contained asphaltenes only, E_AR_ contained an asphaltene/resin mixture, E_AH_ contained an asphaltene/HPAM mixture, E_RH_ contained a resin/HPAM mixture, and E_ARH_ contained the ternary mixture of asphaltenes/resins/HPAM. For the biodegradation series, samples containing the bacterial strain were designated as E_BHx_ groups, and the biodegradation times in the experimental groups (E_BH1_, E_BH3_, E_BH5_, and E_BH7_) were 1, 3, 5, and 7 days, respectively. Correspondingly, cell-free emulsions prepared from the supernatants of the biodegraded systems, which were centrifuged at 10,000× *g* for 10 min to completely remove bacterial cells, were designated as E_CHx_ groups (E_CH1_, E_CH3_, E_CH5_, and E_CH7_) to isolate and quantify the impact of biodegradation metabolites on emulsion interfacial properties, eliminating contributions from bacterial cells. The detailed composition, component concentrations, and group designations of all emulsion systems are summarized in Table S1.

### 2.5. Zeta Potential Measurement

The emulsion was transferred to a disposable folded capillary cell, and the zeta potential was measured by electrophoretic light scattering using a Zetasizer Nano ZS instrument (Malvern Panalytical Ltd., Malvern, UK) at 25 °C. Each measurement was performed in triplicate.

### 2.6. Emulsion Stability Analysis

The prepared emulsion was transferred to a dedicated Turbiscan measurement vial. The Turbiscan Stability Index (TSI) was recorded every 12 h for 4 days at 25 °C using a Turbiscan Lab instrument (Formulaction, Toulouse, France). The TSI, calculated by the instrument software, was used to evaluate the dynamic stability of the emulsions, with higher TSI values indicating lower stability.

### 2.7. Shear Rheological Characterization of Emulsions

The shear rheological properties of the emulsions were characterized using a HAAKE MARS rotational rheometer (Thermo Fisher Scientific, Waltham, MA, USA) equipped with a parallel plate geometry (P35 Ti L-type, 35 mm diameter). All measurements were conducted at 25 °C. For each test, 1 mL of emulsion was carefully loaded onto the lower plate using a micropipette to ensure uniform sample distribution.

Yield stress measurements were performed by conducting stress sweep tests over a shear stress (*τ*) range of 0.1–10 Pa. The yield stress was determined from the critical stress value at which the transition from elastic to viscous behavior occurred.

Dynamic oscillatory measurements were conducted within the frequency range of 0.1–100 Hz at a constant shear stress of 0.25 Pa, which was pre-determined to be within the linear viscoelastic region (LVR) through amplitude sweep tests. The storage modulus (*G*′) and loss modulus (*G*″) were recorded as functions of frequency to characterize the viscoelastic behavior of the emulsions.

### 2.8. Characterization of HPAM

HPAM samples were pre-frozen at an ultra-low temperature for 12 h and subsequently lyophilized for 48 h. The dried residues were characterized using SEM (FEI Verios 460 L, Thermo Fisher Scientific, Waltham, MA, USA), Fourier Transform Infrared Spectroscopy (FT-IR) (IRSpirit, Shimadzu Corporation, Kyoto, Japan) to elucidate the structural and chemical alterations induced by biodegradation. Typical biological metabolites were identified using a TRACE 1300 GC-MS (Thermo Fisher Scientific, Waltham, MA, USA) equipped with a DB-5MS column. The GC oven temperature program was initiated at 80 °C (held for 3 min), ramped to 300 °C at a rate of 10 °C/min, and maintained for 5 min. Helium served as the carrier gas at a flow rate of 1.0 mL/min. The injector temperature and transfer line temperature were set to 300 °C. Samples (1 μL) were injected in split mode (15:1). The mass spectrometer operated in Electron Impact (EI) mode (70 eV) with an ion source temperature of 300 °C, scanning a mass range of 15–550 *m*/*z* with a solvent delay of 2.5 min. Dichloromethane (Merck KGaA, Darmstadt, Germany) was used as the needle wash solvent.

### 2.9. Statistical Analysis

All experiments were repeated at least three times, and the results are expressed as the mean ± standard deviation. Origin 2021 software was used to calculate the mean and standard deviation and to generate the graphs.

## 3. Results and Discussion

### 3.1. Isolation and Identification of the HPAM-Degrading Strain EPDB-8

**Figure 1:**
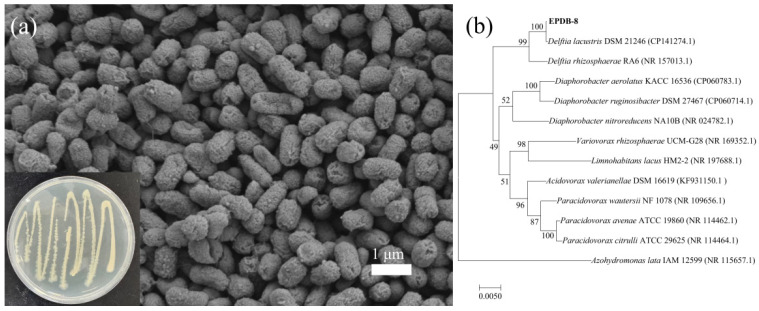
(**a**) Microscopic morphology of strain EPDB-8, (**b**) phylogenetic tree based on 16S rRNA gene sequences showing the relationship between strain EPDB-8 and related taxa.

A eutrophic-to-oligotrophic gradient adaptation strategy was used to enrich microorganisms capable of degrading HPAM. Through stepwise enrichment and screening, a pure strain with efficient HPAM-degrading capability was isolated. The amplified 16S rRNA gene fragment of this strain was 1383 bp in length. NCBI BLAST (MEGA 11.0) analysis showed that the sequence shared high similarity with bacteria of the genus *Delftia*. Further phylogenetic analysis revealed that this strain clustered with *Delftia lacustris* DSM 21246 in the same branch, indicating its closest phylogenetic relationship with *Delftia lacustris*. Therefore, based on the BLAST results and phylogenetic analysis, the strain was identified as *Delftia lacustris* and designated *Delftia lacustris* strain EPDB-8 (Figure 1b). The 16S rRNA gene sequence of this strain has been deposited in GenBank under accession number PZ384026. Morphological characterization showed that EPDB-8 is a rod-shaped bacterium approximately 1 μm in length (Figure 1a). Among the different enrichment strategies tested, the continuous passage cultivation method achieved the highest cell density (Table S2), and the HPAM degradation efficiency reached 90%. The genus *Delftia* is known for its metabolic versatility and its ability to degrade structurally complex organic compounds, which is consistent with the efficient HPAM-degrading capability of strain EPDB-8 observed in this study [21,22].

### 3.2. Changes in Interfacial Properties During Biodegradation

**Figure 2:**
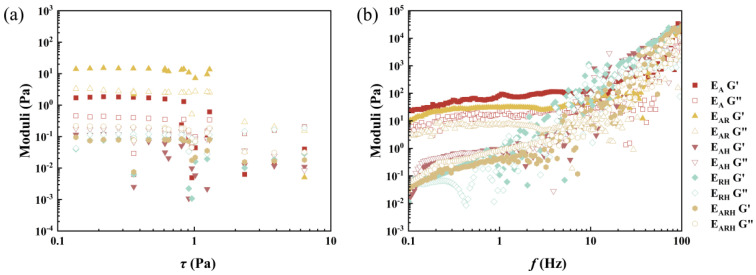
Shear rheological properties of emulsions: (**a**) *τ* dependence, (**b**) ƒ dependence.

To understand the macroscopic flow behavior and stability of the emulsions, the study employed shear rheology and oscillatory measurements. The yield stress, *G*′ and *G*″ were measured for the control emulsion (E_ARH_) and the biodegraded emulsions (E_BH_ series) at various time points. The results show that different stabilization mechanisms correspond to distinct interfacial structures and macroscopic mechanical responses [23,24,25,26]. The E_AR_ emulsion exhibited a dominant elastic behavior across the entire frequency range, with the *G*′ consistently higher than the *G*″ (Figure 2b), indicating solid-like behavior [27]. This high elasticity was attributed to the rigid, glassy, and irreversible adsorption of asphaltenes at the oil–water interface [28], forming a dense interfacial film. However, its yield stress was only about 2.46 Pa (Figure 2a), suggesting that this rigid interface is brittle and prone to rupture once the critical stress is exceeded [29]. In contrast, the E_ARH_ emulsion transitioned into a typical O/W system. Throughout the frequency range, *G*″ was clearly higher than *G*′, demonstrating a viscosity-dominated characteristic. Emulsions stabilized by HPAM behave as viscoelastic fluids rather than simple Newtonian fluids, due to the entangled polymer network formed in aqueous solution. At low shear rates, HPAM chains adopt coiled and entangled conformations that hinder flow and result in high viscosity, while at high shear rates, the chains become stretched and disentangled, leading to shear-thinning behavior typical of polymer solutions, without forming a truly crosslinked elastic network [3,30,31]. Despite the reduction in oscillatory moduli compared to the rigid asphaltene film, the yield stress of E_ARH_ increased markedly, exceeding 10 Pa (Figure 2a). This indicates that HPAM enhances the system’s resistance to yielding, mainly by increasing the viscosity of the aqueous phase, enhancing chain entanglement, and providing steric hindrance and electrostatic repulsion. It forms a more flexible and deformable composite interfacial film that is tougher than the asphaltene film alone.

**Figure 3:**
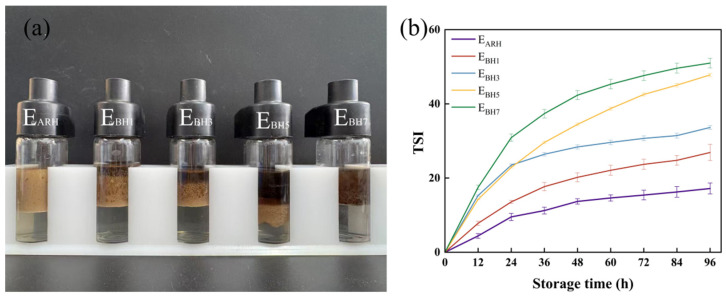
(**a**) Test photos of HPAM degradation for different times, (**b**) TSI of HPAM degradation for different times.

**Figure 4:**
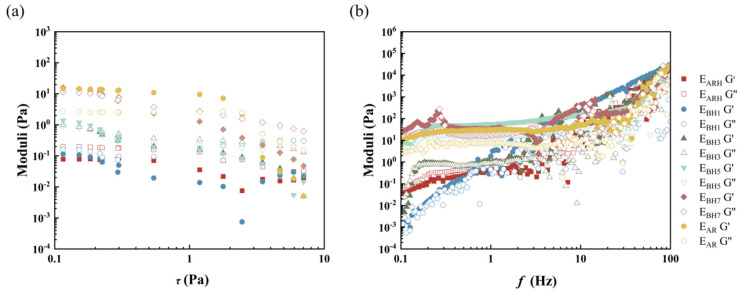
Shear rheological properties of strain-containing emulsions: (**a**) *τ* dependence, (**b**) ƒ dependence.

The degradation of HPAM disrupts its entangled network structure, reducing the confinement on oil droplets and promoting their coalescence and separation, resulting in an increase in oil layer height and TSI over time (Figure 3). As degradation proceeds, the emulsion exhibits a gradual transition from viscous-dominated behavior (*G*″ > *G*′) to elastic-dominated behavior (*G*′ > *G*″), with a concomitant increase in viscoelastic moduli (Figure 4). This evolution indicates the progressive reconstruction of the internal structure, ultimately approaching the rheological properties of the initial E_ARH_ aqueous phase and further recovering toward those of E_AR_.

Overall, the rheological evolution of the emulsions clearly reflects the shift in dominant stabilization mechanism during HPAM biodegradation. In E_ARH_, the enhanced yield stress despite lower oscillatory moduli indicates that emulsion stability is governed primarily by the viscous resistance of the HPAM-rich continuous phase and the toughness of the flexible composite interfacial film, rather than by a rigid elastic interface alone. As HPAM is progressively degraded, the loss of polymer entanglement weakens droplet confinement and reduces resistance to flow and coalescence, which is consistent with the increasing oil layer height and TSI. Meanwhile, the gradual transition from viscous-dominated to elastic-dominated behavior suggests that the system progressively loses polymer-assisted stabilization and reverts toward an asphaltene-controlled interfacial structure.

### 3.3. Role of Bacterial Cells in Emulsion Stabilization

To decouple the biodegradation activity of the strain from the physical contribution of bacterial cells as solid particles to the emulsion network, two control groups were established: E_BH_ (bacterial cells and HPAM) and E_CH_ (equivalent degradation products only, no bacterial cells). Because bacterial particles were absent in E_CH_, differences in rheological behavior and stability between the two groups were attributed primarily to the physical role of the cells.

**Figure 5:**
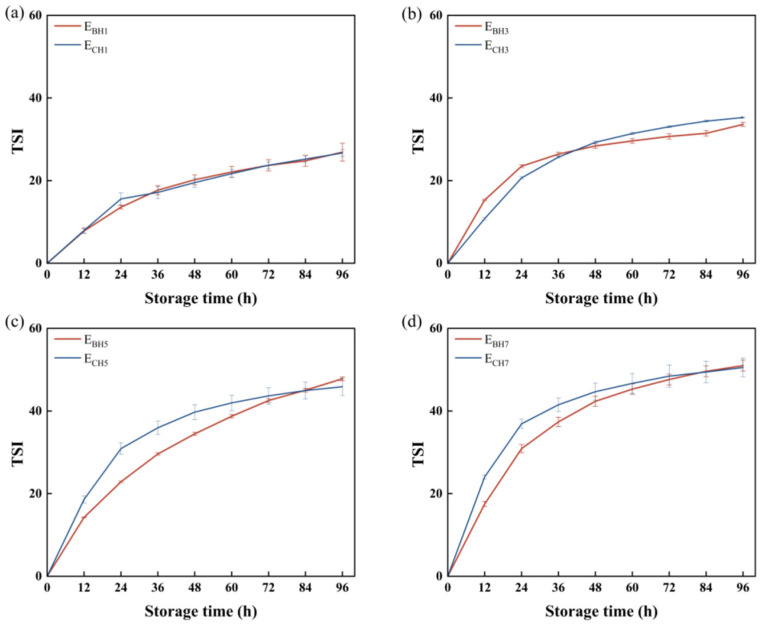
Effect of strains on TSI of emulsions with different degradation days: (**a**) effect of strains on TSI of emulsions at 1 day of degradation, (**b**) effect of strains on TSI of emulsions at 3 days of degradation, (**c**) effect of strains on TSI of emulsions at 5 days of degradation, (**d**) effect of strains on TSI of emulsions at 7 days of degradation.

**Figure 6:**
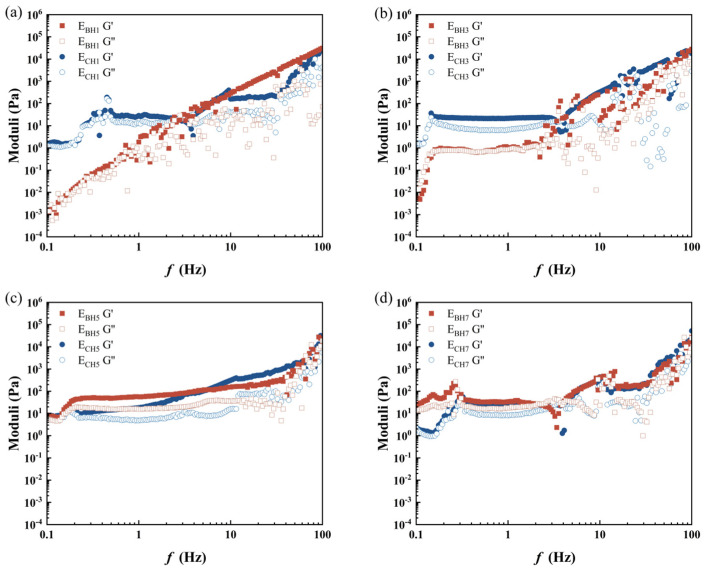
Effect of strains on the shear rheological of emulsions at different degradation days: (**a**) effect of strains on the shear rheological of emulsions at 1 day of degradation, (**b**) effect of strains on the shear rheological of emulsions at 3 days of degradation, (**c**) effect of strains on the shear rheological of emulsions at 5 days of degradation, (**d**) effect of strains on the shear rheological of emulsions at 7 days of degradation.

During the early degradation period (days 1 to 3, Figure 5a,b), the TSI profiles of E_BH_ and E_CH_ were nearly superimposable, with highly consistent growth trends over 96 h, indicating negligible contribution of bacterial particles to macroscopic emulsion stability at this stage. Rheologically, E_CH_ exhibited higher overall *G*′ and *G*″ values with weaker frequency dependence than E_BH_, suggesting that residual HPAM chains, in the absence of cellular interference, retained a relatively continuous three-dimensional network capable of conferring greater structural rigidity and perturbation resistance [32,33,34] (Figure 6a,b). The lower moduli observed in E_BH_ indicated that bacterial particles provided no measurable network reinforcement; rather, their presence likely disrupted interfacial film integrity and network continuity, implicating biodegradation, not particle-mediated physical reinforcement, as the dominant mechanism of the strain at this stage. A premature upturn in *G*′ and *G*″ at high frequencies in E_BH_ further reflected reduced network homogeneity, wherein the mechanical response was governed increasingly by local structural units at shorter relaxation timescales. The appearance of a local *G*′ peak in the low-frequency region, accompanied by a more pronounced low-frequency transition relative to the E_ARH_ group, indicated the presence of characteristic relaxation processes associated with multi-scale heterogeneous structures or rearrangement of locally weakened network domains, features particularly susceptible to low-frequency perturbations [31,35].

As degradation extended to days 5 to 7 (Figure 5c,d), the TSI profiles of E_BH_ and E_CH_ diverged markedly. The TSI of E_CH_ increased rapidly, approaching or exceeding 40 within 96 h, indicative of accelerated destabilization. By contrast, the TSI of E_BH_ rose more gradually and remained substantially lower than that of E_CH_ over the same period, with the most pronounced divergence observed on day 5, coinciding with peak cell density (*OD*_600_ ≈ 2.5, Figure S1). In E_CH_, the combination of HPAM degradation and the absence of compensating particles led to rapid collapse of interfacial protection and consequent demulsification [36,37]. In E_BH_, intact bacterial cells adsorbed at the oil–water interface in a Pickering-like fashion, partially offsetting the loss of the polymeric interfacial film and temporarily sustaining interfacial mechanical strength. This interpretation was corroborated by the rheological data, in which E_BH_ displayed higher yield stress and a more pronounced elastic response at this stage, confirming that bacterial particle adsorption exerted measurable effects on both macroscopic stability and microscopic viscoelastic behavior (Figure 6c,d).

Across all groups, the moduli decreased by approximately one order of magnitude relative to initial values, reflecting progressive scission of HPAM chains and the consequent deterioration of the elastic network backbone [14,38]. Concurrently, the low-frequency moduli of E_BH_ gradually approached and partially exceeded those of E_CH_, indicating that the long-timescale viscoelastic response of E_BH_ was no longer governed by the original HPAM network but was instead shaped by heterogeneous residual structures and bacterial particle components formed upon degradation. Nevertheless, by day 7, the TSI values of both groups continued to rise and converged during prolonged storage, ultimately exhibiting clear destabilization. This convergence demonstrated that, once the polymer network was extensively disrupted, bacterial particle-mediated interfacial protection was insufficient to sustain long-term emulsion stability. The stabilizing contribution of bacterial particles therefore represents a transient compensatory effect rather than a fundamental substitute for the structural role of the polymer network. To further elucidate the mechanistic role of the microorganism in this system, the HPAM degradation products were subsequently characterized in detail.

### 3.4. Role of HPAM Biodegradation in Emulsion Destabilization

**Figure 7:**
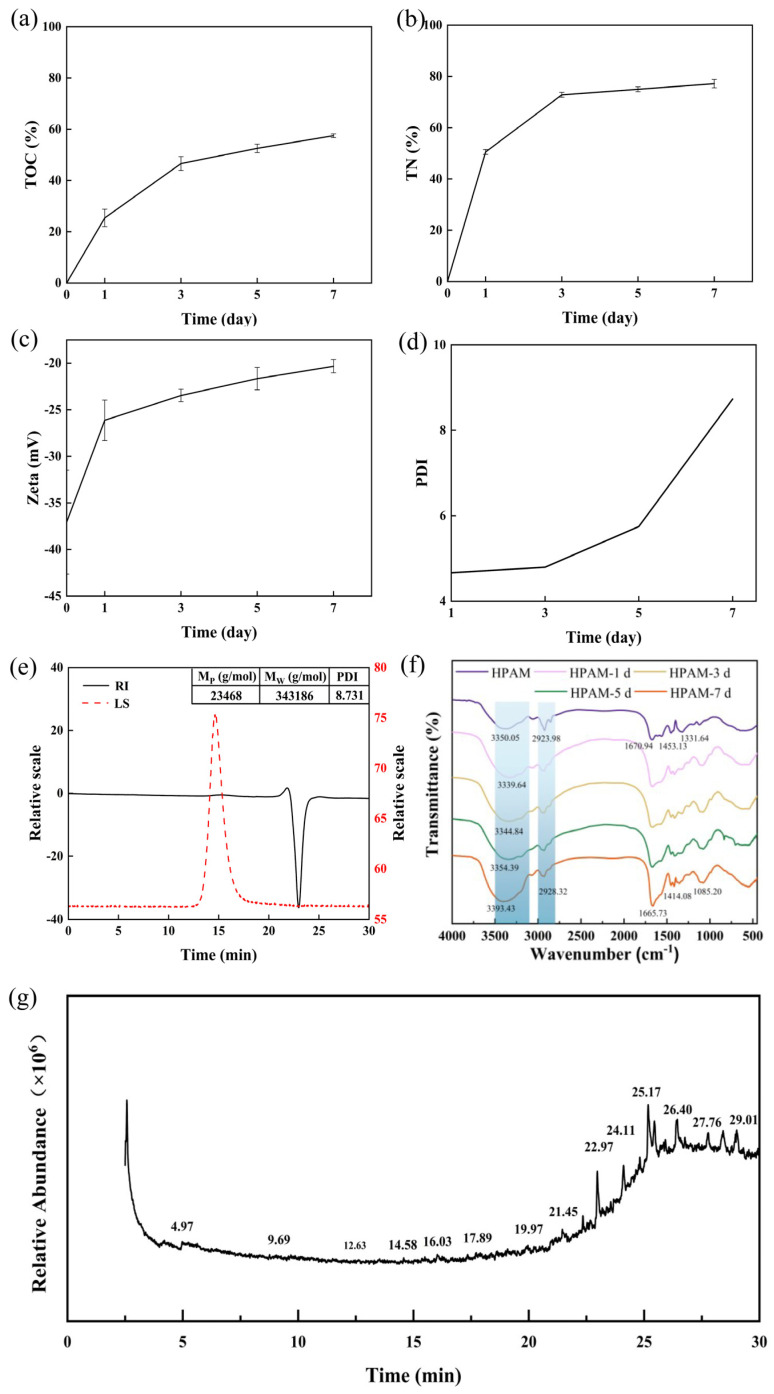
(**a**) TOC removal rate for different days of HPAM degradation, (**b**) TN removal rate for different days of HPAM degradation, (**c**) Zeta potential for different days of HPAM degradation, (**d**) PDI for different days of HPAM degradation, (**e**) GPC chromatograms of HPAM before and after 7 days of biodegradation, (**f**) FT-IR spectra of HPAM degradation for different days and (**g**) GC-MS spectrum of typical biological metabolites produced by strain EPDB-8.

To evaluate degradation efficacy, EPDB-8 was cultivated for 7 days in a mineral salt medium containing 500 mg/L HPAM as the sole carbon and nitrogen source. Degradation performance was assessed via TOC and TN analyses at regular intervals (days 1, 3, 5, and 7). The 500 mg/L concentration was selected to simulate typical residual polymer levels in produced water following polymer flooding. While injected concentrations can exceed 1500–2000 mg/L, adsorption and retention within the reservoir typically dilute the produced concentration to the 200–1000 mg/L range [39]. The TOC removal rate increased progressively with incubation time, reaching 62.93% by day 7 (Figure 7a), indicating that EPDB-8 continuously assimilated carbon-containing fragments released from HPAM degradation throughout the incubation period. The TN removal rate reached 77.71% by day 7 (Figure 7b), reflecting substantial hydrolysis of amide functional groups and confirming the nitrogen-utilizing capacity of strain EPDB-8. Notably, TN removal consistently exceeded TOC removal at each time point, suggesting that nitrogen-containing moieties were preferentially mobilized relative to the carbon backbone. This differential removal pattern implies that EPDB-8 preferentially targets amide side chains prior to attacking the C-C backbone, consistent with a deamidation-dominant degradation strategy. It is well documented that amide side chains are chemically more labile than the carbon backbone, and many bacteria preferentially hydrolyze nitrogen-containing groups before degrading the carbon skeleton [17,18,38,40]. The released ammonia likely serves as a readily assimilable nitrogen source supporting bacterial growth (Table S3), which in turn fuels the production of backbone-degrading enzymes.

HPAM is an anionic polyelectrolyte due to the presence of carboxyl groups formed during partial hydrolysis. In produced water, oil droplets are also typically negatively charged [41,42]. The stability of the emulsion relies heavily on the electrostatic repulsion generated by the adsorbed layer of anionic HPAM, which prevents droplet coalescence. The ζ-potential is a proxy for the magnitude of this repulsive force. A high absolute value (e.g., −40 mV) implies a stable suspension where particles repel each other. Zeta potential measurements were conducted to assess the surface charge characteristics of the polymer/bacterial suspension over 7 days (Figure 7c). Throughout the incubation period, all measured ζ-potential values remained negative, indicating that HPAM-stabilized oil droplets retained anionic surface charges during biodegradation [43]. However, the absolute ζ-potential value decreased progressively with incubation time. This decline can be attributed to EPDB-8-mediated cleavage of amide side chains and the polymer backbone, generating lower-molecular-weight fragments that adsorb less effectively at the oil–water interface. Consequently, the protective anionic polymer layer surrounding oil droplets became thinner, reducing surface charge density and electrostatic repulsion. As the absolute ζ-potential approached zero, the electrical double layer became compressed, lowering the repulsive energy barrier between droplets [44]. According to DLVO theory, this allows attractive van der Waals forces to dominate at short range, promoting droplet aggregation and coalescence and ultimately destabilizing the emulsion [13,45].

GPC was used to track molecular-weight distribution (*Mw*, *Mn*) and polydispersity (PDI) over the incubation period (days 0–7, Figure 7d,e). By day 7, *Mw* decreased from 2.16 × 10^7^ to 3.43 × 10^5^ g/mol. This represents a nearly 63-fold reduction in chain length. The efficacy of HPAM as a viscosifier is directly proportional to its hydrodynamic volume [37], which is a function of its molecular weight (typically 10^7^ Da). A reduction in *Mw* is the proof of backbone scission. Without scission, a polymer might lose viscosity due to conformational collapse (e.g., salinity effects) without actually degrading. GPC distinguishes actual degradation from conformational changes. The PDI progressively increased over time, reaching 8.731 by day 7. The increase in PDI is consistent with a random scission process during degradation. This trend suggests that chain cleavage occurred at multiple positions along the backbone, broadening the molecular weight distribution. Furthermore, the peak molecular weight of the degraded polymer was found to be 2.34 × 10^4^ g/mol, indicating the accumulation of oligomers. These results confirm the significant depolymerization capacity of strain EPDB-8 toward HPAM. These results indicate extensive chain scission of HPAM by strain EPDB-8, accompanied by the accumulation of lower-molecular-weight products. Although TOC, TN, and GPC can be used to obtain elemental and stoichiometric assessments, the chemical transformation pathways of the polymer remain to be elucidated.

The progressive chemical transformations described above are expected to exert profound effects on emulsion stability through two interrelated mechanisms. First, chain scission and deamidation occurring in the early stage of degradation (day 1) would significantly reduce the viscosity of the continuous phase and disrupt the entangled polymer network that provides bulk structural support for the emulsion, thereby weakening both the interfacial film strength and the bulk resistance to droplet movement. As degradation proceeds into the intermediate stage, bacterial cells and their degradation products may act as particulate stabilizers in a Pickering-like manner during the degradation process. However, once HPAM is largely degraded, this cell-enriched interfacial layer alone is no longer sufficient to maintain the macroscopic stability of the emulsion. Nevertheless, the intrinsic relationship between this interfacial stabilization effect and the biodegradation of HPAM, as well as their respective contributions, remains to be further clarified.

### 3.5. Structural Changes in HPAM During Biodegradation

**Figure 8:**
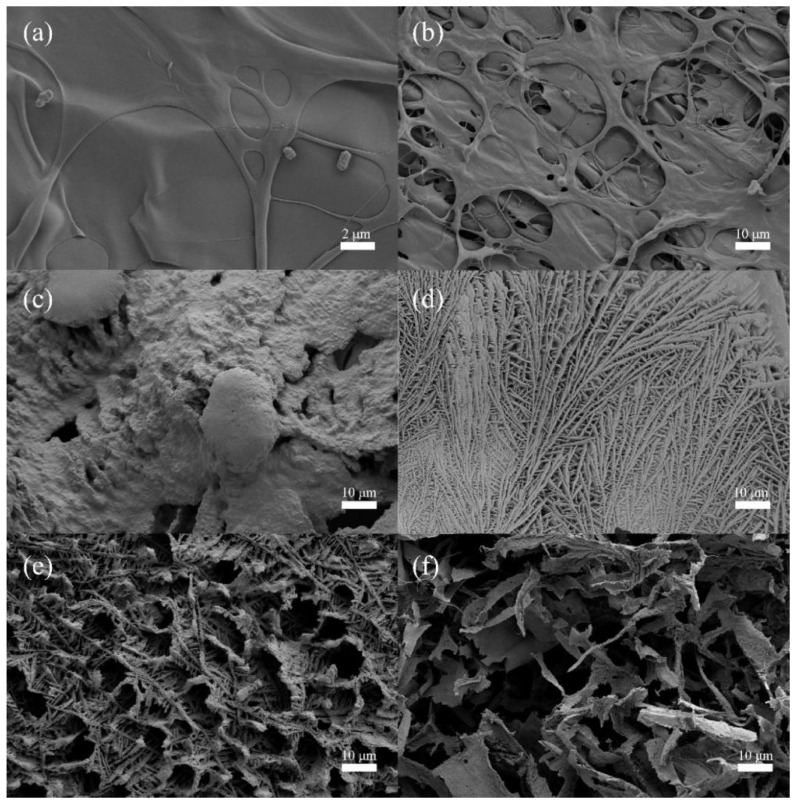
(**a**) SEM image of strains with HPAM, (**b**) SEM image before HPAM degradation, (**c**) SEM image of 1 day of HPAM degradation, (**d**) SEM image of 3 days of HPAM degradation, (**e**) SEM image of 5 days of HPAM degradation, (**f**) SEM image of 7 days of HPAM degradation.

SEM was used to monitor the microscopic integrity of the HPAM polymer network during biodegradation (days 0, 1, 3, 5, and 7). Over the 7 days incubation period, SEM images showed a progressive morphological transformation of the HPAM surface from a relatively smooth and porous structure to a fragmented and collapsed network (Figure 8), consistent with ongoing biodegradation by strain EPDB-8. These observations suggest that bacterial attack likely initiated from surface attachment (Figure 8a) and then gradually progressed inward (Figure 8b–f), leading to continuous erosion of the polymer matrix [43,44]. To investigate the functional group changes in HPAM during the biological treatment by strain EPDB-8, FT-IR analysis was performed on HPAM samples at different degradation times (Figure 7f). After 1 day of degradation, the amide N-H stretching vibration (3350 cm^−1^) exhibited a red shift (3344 cm^−1^), the methyl C-H stretching vibration (2924 cm^−1^) broadened and shifted to lower wavenumbers, which, combined with the attenuation of the C-N bending vibration, is consistent with scission of the HPAM carbon backbone [45]. The resulting lower-molecular-weight fragments may be more accessible for microbial utilization by strain EPDB-8. Furthermore, the red shift in the C = O stretching vibration (1671 cm^−1^) and the emergence of a new C-O stretching vibration (1085 cm^−1^) suggest the formation of additional oxygen-containing functionalities during biodegradation [46,47]. Together with the GPC results, the FT-IR data support the view that EPDB-8 transforms HPAM through side-group conversion and chain scission, consistent with the observed TOC and TN reductions.

Additionally, GC-MS was used to qualitatively characterize the degradation products present in the supernatant after 7 days. Based on tentative library matching (SI~700–750), the supernatant indicated the possible presence of long-chain fatty acid amides (e.g., oleamide, palmitoleamide, and cis-11-eicosenamide, Figure S3a–h). Compounds of this type have been reported in *Delftia*-related metabolic profiles and, because of their amphiphilic nature, may influence oil–water interfacial behavior [48,49,50,51]. These long chain fatty acid amides exhibit quorum sensing regulation, biosurfactant properties, and transboundary signaling capabilities [50]. In addition to oleamide, the study also identified palmitoleamide, linoleamide, and stearamide in the metabolites of *Delftia lacustris*. In the present study, the detection of hexadecenenitrile and 9-octadecenenitrile in Figure S3 was consistent with the occurrence of C16- and C18-amide-related compounds in the degradation system [51], suggesting that, in addition to polymer chain scission and functional-group transformation, the biodegradation process may also involve the generation of amphiphilic metabolites that contribute to interfacial modulation. These metabolites may help bacteria access and utilize insoluble carbon sources. Separately, although oleamide and erucamide are known plastic slip agents, the simultaneous detection of non-additive compounds such as 17-pentatriacontene and pregnan-20-one indicates a biological rather than artifactual origin.

### 3.6. Mechanistic Model of Biodegradation Induced Demulsification

**Figure 9:**
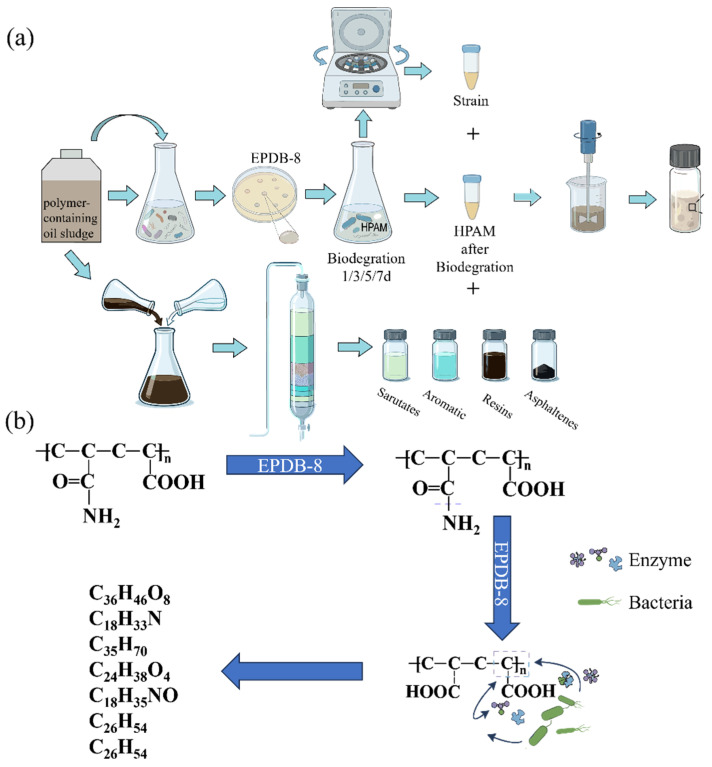
Schematic illustration of the overall research strategy and the proposed mechanism of HPAM biodegradation. (**a**) Overview of the research workflow. (**b**) Proposed schematic and mechanistic model of HPAM biodegradation by EPDB-8.

Combining the above observations, a staged mechanistic model is proposed to explain how HPAM biodegradation by *Delftia lacustris* EPDB-8 progressively weakens oil-in-water emulsions. The evolution of emulsion stability is governed primarily by changes in polymer integrity and interfacial mechanical properties, with microbial biomass acting as a transient secondary modulator (Figure 9).

Upon inoculation, EPDB-8 may interact with the HPAM-conditioned oil–water interface. Early biodegradation is characterized by partial deamidation and limited chain scission, while high molecular weight HPAM still dominates the interfacial and bulk phases. As a result, the emulsion remains largely intact, although slight weakening is observed, reflected by a modest increase in TSI and a reduction in yield stress. At this stage, polymer mediated steric and electrostatic stabilization remains the primary resistance to coalescence. As biodegradation proceeds, substantial fragmentation of HPAM reduces polymer chain length and entanglement, leading to an overall decrease in polymer supported viscosity and structural rigidity. Concurrently, the accumulation of bacterial cells at the interface and within the continuous phase becomes significant. These cell-associated structures, potentially together with extracellular materials produced during growth, may contribute to a heterogeneous interfacial layer that temporarily bears mechanical stress. This transition explains the non-monotonic rheological response observed experimentally, where *G*′ and *G*″ partially recover and yield stress increases during this period. The resulting interface resembles a transient, bio-assisted particulate film rather than a continuous polymeric layer, providing limited but measurable resistance to deformation without restoring long-term stability. By day 7, HPAM is extensively degraded, and the contribution of polymer chains to interfacial cohesion and bulk phase viscosity becomes negligible. The loss of the polymer scaffold eliminates the dominant steric and viscoelastic barrier preventing droplet coalescence, resulting in a sharp decrease in yield stress and rapid emulsion breakdown. Although bacterial cells and associated materials may still contribute to short-term elastic responses under small deformations, such interfaces lack the continuity and durability required for sustained stabilization, ultimately leading to phase separation. This interpretation is consistent with the observed evolution of TSI, yield stress, and viscoelastic moduli, and highlights polymer integrity as the controlling factor in the long-term stability of HPAM stabilized emulsions.

It is also important to note that several low-molecular-weight products tentatively identified by GC-MS, such as oleamide, may influence interfacial behavior and could contribute to the observed non-linear rheological trends.

## 4. Conclusions

This study establishes a mechanistic framework linking HPAM biodegradation, bulk and interfacial rheological evolution, and emulsion destabilization by *Delftia lacustris* EPDB-8. The strain efficiently destabilized HPAM-stabilized oil-in-water emulsions through a coupled mechanism of polymer depolymerization and interfacial mechanical weakening. Cultivated in mineral salt medium with 500 mg/L HPAM as the sole carbon and nitrogen source, EPDB-8 achieved TOC and TN removal rates of 62.93% and 77.71%, respectively, within 7 days. Enzymatic deamidation and random chain scission progressively disrupted the polymer architecture, collapsing the entanglement network and attenuating bulk viscoelasticity. Interfacial characterization further demonstrated that the depletion of high-molecular-weight chains dismantled the viscoelastic composite interfacial film co-formed by HPAM and indigenous surface-active species, shifting the dominant stabilization mechanism from polymer-assisted reinforcement to a less effective asphaltene-dominated structure and thereby accelerating droplet coalescence and macroscopic phase separation. Although bacterial cells exerted a transient particle-stabilization effect at the oil–water interface, long-term emulsion stability was governed primarily by polymer integrity rather than by the presence of suspended bioparticles.

By simultaneously collapsing the continuous-phase viscoelastic network and weakening the interfacial film, EPDB-8 facilitates the release of entrapped crude oil from otherwise intractable polymer-stabilized emulsions. These findings offer a safe and mechanistically grounded biological demulsification strategy for the treatment of polymer-flooding produced water. Practical deployment could be achieved through bioaugmentation of existing treatment facilities or integration of dedicated aerobic bioreactors upstream of gravity separators or dissolved air flotation units, providing a cost-effective pretreatment step that reduces residual polymer concentration, promotes oil–water separation, and improves both crude oil recovery efficiency and produced-water discharge quality. Future work should focus on optimizing inoculation conditions, evaluating performance under field-relevant salinity and temperature ranges, and assessing the long-term operational stability of continuous bioreactor configurations.

## Figures and Tables

**Table 1:** Representative HPAM Wastewater Characteristics Reported in Published Studies.

Region	Wastewater Type	HPAM (mg/L)	COD	Oil	Suspended Solids	Surfactant	Refs.
China, Daqing oilfield	strong-alkali ASP flooding produced water	635.51–1021.83	2174.82–4144.85	35.01–2205.88	40.00–206.25	190.8–296.8	[6]
China, Shengli oilfield	polymer-flooding produced water	NR	476.63	NR	NR	NR	[7]
China, Xinjiang oilfield	polymer-flooding produced water	176.9–177.1	NR	128–7364	153–990	35.5–43.8	[8]
China, Daqing oilfield	polymer-flooding produced water	100–600	NR	NR	NR	NR	[9]
Oman, Nimr constructed wetland	oilfield produced water	0–1000	NR	NR	microbial mats present	NR	[10]
Oman oilfield	polymer-flooding produced water	352–800	NR	75–450	NR	NR	[11]

Note: NR = not reported in the original article; The original article reported mean ± standard deviation, and the approximate range here was calculated as mean ± SD; it does not represent the actual minimum–maximum range.

## Data Availability

The original contributions presented in this study are included in the article/Supplementary Material. Further inquiries can be directed to the corresponding author.

## Supplementary Materials

The following supporting information can be downloaded at: https://www.mdpi.com/article/10.3390/polym18111268/s1. Figure S1: Growth and developmental curves of strain EPDB-8; Figure S2: HPAM degradation rate at different days determined by the starch–cadmium iodide method; Figure S3: Typical organic metabolites detected in the degradation supernatant of strain EPDB-8: (a) oleamide; (b) pal-mitoleamide; (c) linoleamide; (d) stearamide; (e) cis-11-eicosenamide; (f) hexadecenenitrile; (g) 9-octadecenenitrile; (h) 17-pentatriacontene; Table S1: Emulsion preparation conditions and group designations; Table S2: Cell densities obtained under different enrichment strategies during isolation of HPAM-degrading bacteria; Table S3: Physiological and biochemical characterization of strain EPDB-8; Text S1: HPAM degradation rate measurement using the starch–cadmium iodide method; Text S2: TOC measurement; Text S3: TN measurement. The Supporting Information contains 11 pages, 3 methods, 3 figures, and 3 tables.
