# Supplementary material for: Rheology and Oil–Water Emulsion Stability During Biodegradation of Hydrolyzed Polyacrylamide by Delftia lacustris EPDB-8
Source: Polymers (Basel). 2026 May 22;18(11):1268. doi: 10.3390/polym18111268 (PMC13259527; doi:10.3390/polym18111268)
Supplement: Supplementary file 1 [file polymers-18-01268-s001.zip › polymers-4295351-supplementary.pdf]

Rheology and Oil-Water Emulsion Stability during Biodegradation of HPAM by *Delftia lacustris* EPDB-8

Bingjian Sun[1,2,†], Yanshuo Li[1,2,†], Wei Liu[3], Xin Hu[1,2], Shichong Guo[1,2], Yiming Li[1,2], Jinren Lu[2], Haoshuai Li[1,2], Mutai Bao[1,2,*]

Supporting Information

11 Pages, 3 Methods, 3 Figures, 3 Tables

[1] *Frontiers Science Center for Deep Ocean Multispheres and Earth System, and Key Laboratory of Marine Chemistry Theory and Technology, Ministry of Education, Ocean University of China, Qingdao 266100, China*

[2] *College of Chemistry and Chemical Engineering, Ocean University of China, Qingdao 266100, China*

[3] *Key Laboratory of Colloid and Interface Chemistry of the Ministry of Education, and School of Chemistry and Chemical Engineering, Shandong University, Jinan 250100, China*

[*]Corresponding Author: Mutai Bao. Address: College of Chemistry and Chemical Engineering, Ocean University of China, Songling Road 238, Qingdao 266100, Shandong Province, China. Telephone: 86–0532–66782509; E–mail address: mtbao@ouc.edu.cn.

†B. S. and Y. L. contributed equally to this work.

Text. S1 HPAM degradation rate measurement using the starch cadmium iodide method

The degradation rate of HPAM was determined using the commonly used starch-cadmium iodide method in oilfields. First, 5 mL of acetate buffer solution, 1 mL of the sample to be tested, 20 mL of deionized water, and 1 mL of saturated bromine water were added. The reaction was shaken for 18 minutes, then 5 mL of sodium formate was added, and the reaction was shaken for another 10 minutes. Finally, 5 mL of starch-cadmium iodide solution was added, and the mixture was thoroughly shaken and allowed to develop color for 18 minutes. The absorbance of the solution was measured at a wavelength of 585 nm.

Text. S2 TOC measurement

The total organic carbon (TOC) concentration in the water samples was measured using a TOC analyzer. The TOC sample bottle was heated in a muffle furnace at 550°C for 3 hours to remove residual organic carbon. The sample was then diluted 100 times, and 20 mL of the diluted sample was added to the TOC sample bottle for analysis in the TOC analyzer.

Text. S3 TN measurement

The total nitrogen (TN) concentration in the water samples was measured using a TN reagent tube and a digestion apparatus. The sample was diluted 100 times with nitrogen-free deionized water. The digestion apparatus was heated to 105°C, and a total nitrogen peroxysulfate reagent powder was added to the reagent tube. 0.5 mL of the diluted sample was then added. The reagent tube was vigorously shaken for 30 seconds and placed into the digestion apparatus at 105°C for 30 minutes. After digestion, the tube was quickly removed and allowed to cool. After cooling, a total nitrogen A reagent powder was added to the tube, which was shaken for 15 seconds, followed by a 3-minute reaction. Total nitrogen B reagent powder was then added to the tube, shaken for 15 seconds, and reacted for 2 minutes. A 2 mL aliquot of the digested total nitrogen solution was transferred to a total nitrogen C reagent tube. The total nitrogen C reagent tube was gently inverted 10 times and reacted for 5 minutes. The water quality multi-parameter instrument (e.g., DR3900, Hach, USA) was set to the total nitrogen HR TNT test program. The blank reagent tube was wiped clean and placed into the 16 mm round adapter of the multi-parameter instrument for calibration. The reagent tube containing the sample was then wiped clean and placed into the instrument for total nitrogen content measurement.

Supplementary Figures

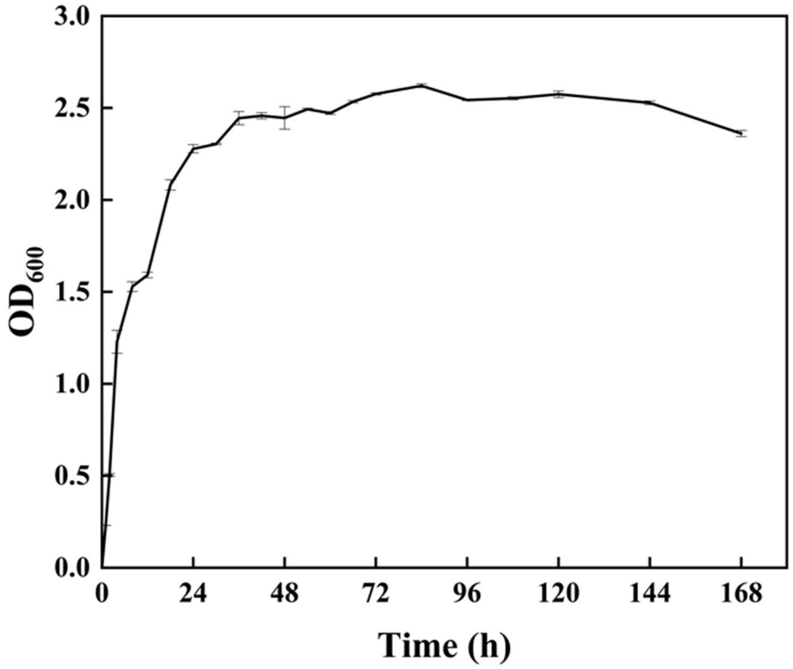

Figure S1 Growth and developmental curves of strain EPDB-8

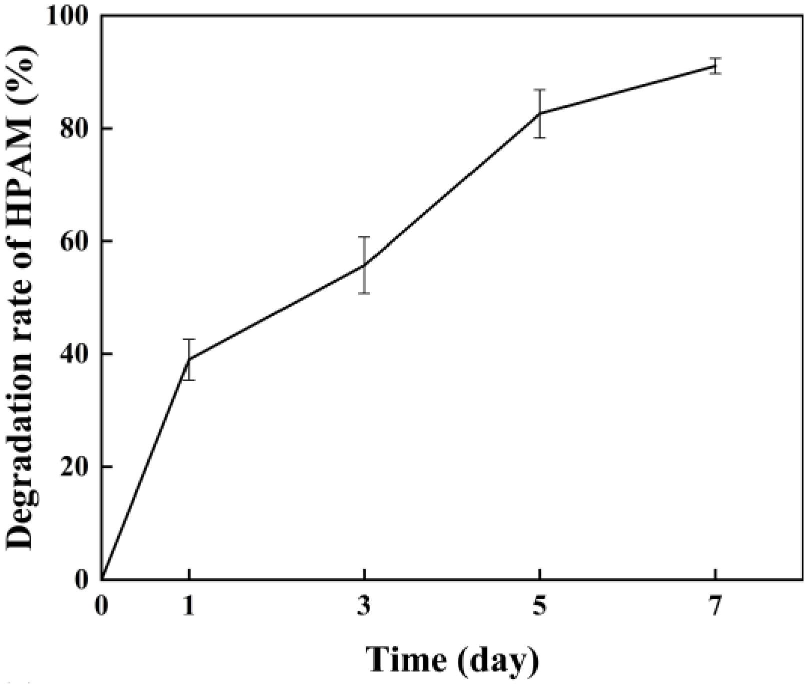

Figure S2. HPAM degradation rate at different days determined by the starch-cadmium iodide method

(a)

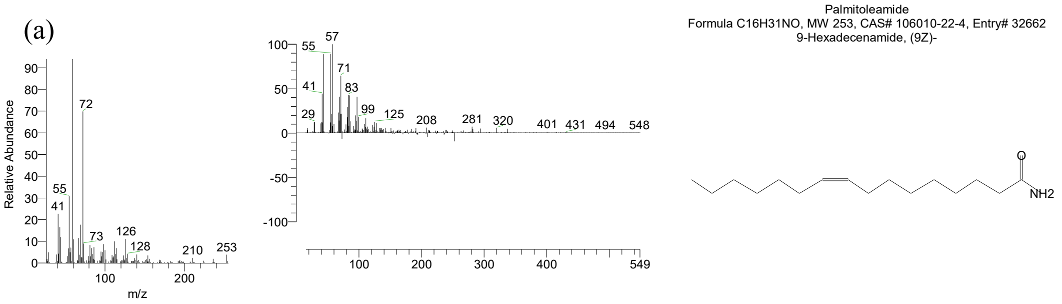
Palmitoleamide
Formula C16H31NO, MW 253, CAS# 106010-22-4, Entry# 32662
9-Hexadecenamide, (9Z)-
Relative Abundance
m/z
NH2
O

(b)

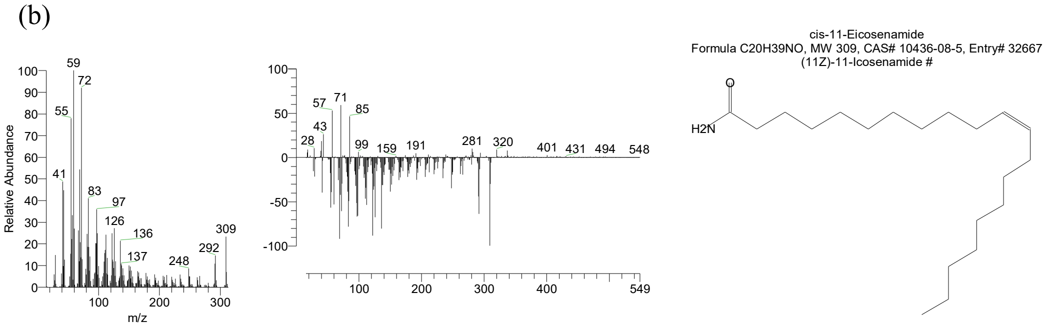
cis-11-Eicosenamide
Formula C20H39NO, MW 309, CAS# 10436-08-5, Entry# 32667
(11Z)-11-Icosenamide #
Relative Abundance
m/z
H2N
O

(c)

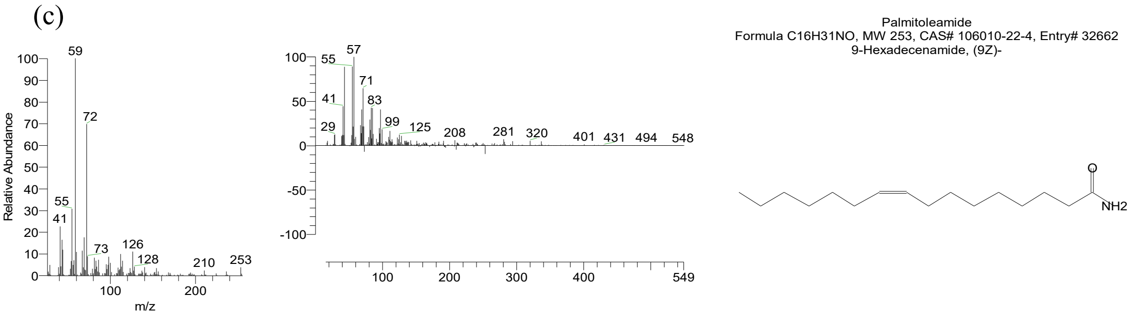
Palmitoleamide
Formula C16H31NO, MW 253, CAS# 106010-22-4, Entry# 32662
9-Hexadecenamide, (9Z)-
Relative Abundance
m/z
NH2
O

(d)

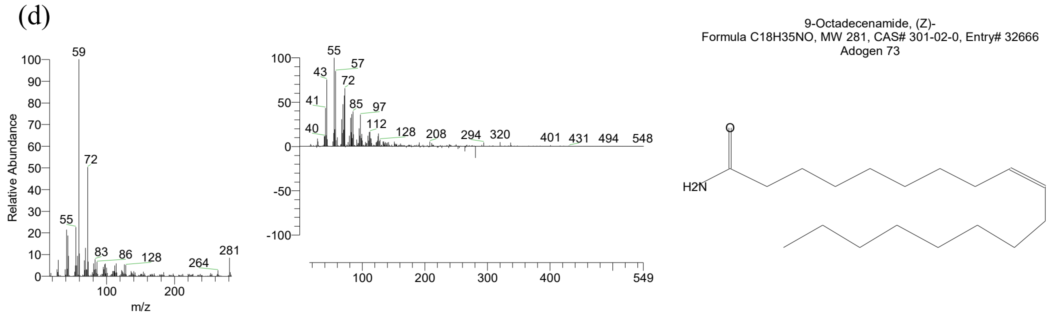
9-Octadecenamide, (Z)-
Formula C18H35NO, MW 281, CAS# 301-02-0, Entry# 32666
Adogen 73
Relative Abundance
m/z
H2N
O

(e)

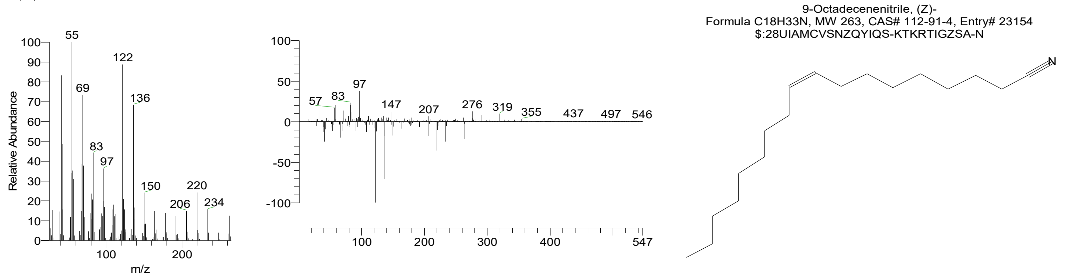

(f)

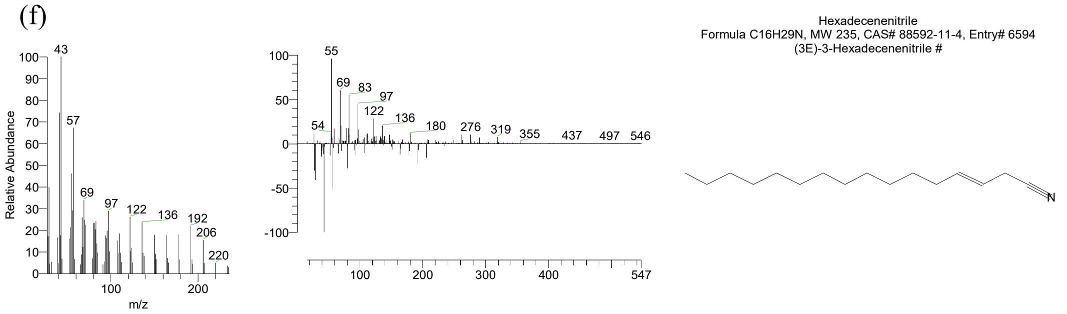

(g)

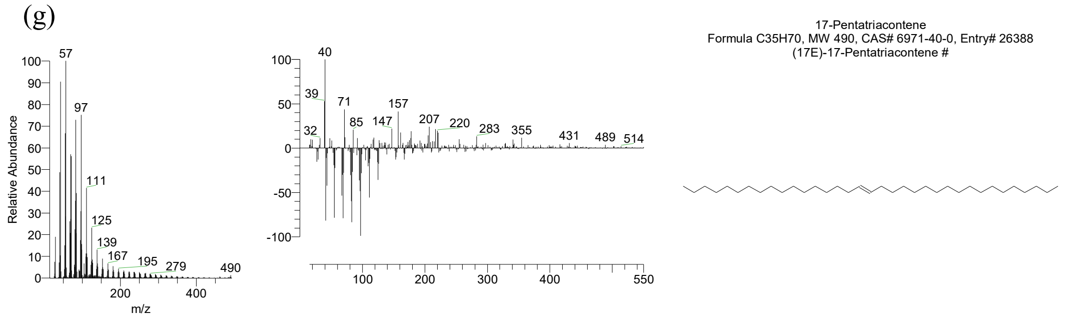

(h)

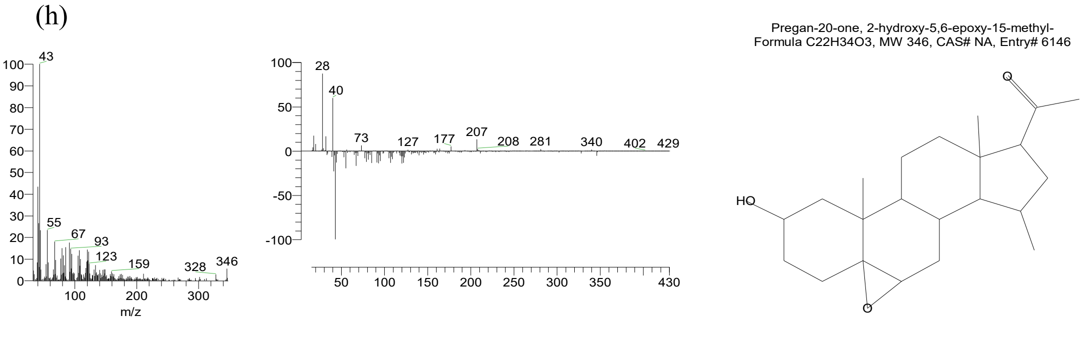

Figure S3. Figure S3: Typical organic metabolites detected in the degradation supernatant of strain EPDB-8: (a) oleamide; (b) palmitoleamide; (c) linoleamide; (d) stearamide; (e) cis-11-eicosenamide; (f) hexadecenenitrile; (g) 9-octadecenenitrile; (h) 17-pentatriacontene.

Supplementary Table

Table.S1 Emulsion preparation conditions and group designations

Parameter	Detail
Oil phase	Heptol (toluene/n-heptane, 60:40 v/v)
Aqueous phase	Water
Phase ratio	1:1 (v/v), 5 mL each
Homogenization	10,000 rpm, 2 min
Asphaltene concentration	0.5 g/L in heptol
Resin concentration	0.5 g/L in heptol
Equilibration	6 h quiescent standing before analysis

Group	Detail	Notes
EA	Asphaltenes	Control
EAR	Asphaltenes + resins	Control
EAH	Asphaltenes + HPAM	Control
ERH	Resins + HPAM	Control
EARH	Asphaltenes + resins + HPAM	Control
EBH1/3/5/7	EARH + bacteria	Biodegradation at 1, 3, 5, 7 days
ECH1/3/5/7	EARH +Cell-free supernatant	Centrifuged at 10,000 × g, 10 min to remove cells; isolates metabolite contribution

Table S2 Cell densities obtained under different enrichment strategies during isolation of HPAM-degrading bacteria

HPAM (mg/L)	Transfer (log10 (CFU/mL), mean ± SD, n)	Cumulative total dose (log10 (CFU/mL), mean ± SD, n)
50	9.199 ± 0.125 (n=3)	9.604 ± 0.840 (n=3)
100	9.671 ± 0.254 (n=3)	9.522 ± 0.387 (n=3)
150	9.687 ± 0.449 (n=3)	9.241 ± 0.142 (n=3)

Table S3 Physiological and biochemical characterization of strain EPDB-8

Test	EPDB-8
Gram staining	Negative
Spore staining	Negative
Catalase test	Positive
Methyl red (MR) test	Negative
Voges–Proskauer (VP) test	Negative
Starch hydrolysis	Positive
Cellulose hydrolysis	Negative
Gelatin liquefaction	Negative
Amidase test	Positive
